# Comprehensive Phenolic Profiling, Antioxidant Bioactivity, and Pomological Evaluation of Wild Rosehip (*Rosa* spp.) Samples

**DOI:** 10.3390/molecules31193429

**Published:** 2026-09-26

**Authors:** Erol Tunca, Ali Murat Kesemen, Meryem Kara, Aziza Huseynova, Sevgi Kolaylı

**Affiliations:** 1 Department of Chemistry, Faculty of Science, Karadeniz Technical University, Trabzon 61080, Türkiye; eroltunca@ktu.edu.tr; 2 Bee and Bee Products Application and Research Center, Karadeniz Technical University, Trabzon 61080, Türkiye; 3 Artvin Vocational School, Artvin Çoruh University, Artvin 08000, Türkiye; alimuratkesemen@artvin.edu.tr; 4 Çumra Vocational School, Selcuk University, Konya 42500, Türkiye; meryemkara@selcuk.edu.tr; 5 Department of Biochemistry, Faculty of Medicine, Nakhchivan State University, Nakhchivan AZ7000, Azerbaijan; azizahuseynova@ndu.edu.az

**Keywords:** rosehip, antioxidant, phenolic, ellagic acid, ascorbic acid, pomology

## Abstract

Wild rosehip (*Rosa* spp.) fruits are recognized as rich sources of bioactive compounds; however, their quality traits and phenolic profiles can vary considerably. In this study, five composite samples representing four wild *Rosa* taxa, identified and classified based on botanical morphological traits, collected from Artvin, Türkiye, were evaluated for pomological characteristics, color (L*, a*, b*), total soluble solids, ascorbic acid, total phenolics (TPC), total flavonoids (TFC), antioxidant capacity (DPPH and FRAP), and targeted phenolic profiles via HPLC–PDA screened against 26 phenolic standards. Significant differences were observed among the analyzed composite samples. Ascorbic acid, TPC, and TFC ranged from 78 to 211 mg/100 g FW, 905 to 2902 mg/100 g FW, and 115 to 510 mg/100 g FW, respectively, with FRAP values spanning 1.71–4.39 µmol Trolox/g FW and DPPH (SC_50_) values of 3.89–17.15 mg/mL. Among the compounds, six were detected and quantified, with ellagic and gallic acids present across all samples, whereas catechin and epicatechin showed marked sample-specific distribution. Notably, samples morphologically identified as *R. pimpinellifolia* (RS4 and RS5) exhibited the highest phenolic content and antioxidant capacity. These results establish baseline quality data for wild rosehips, demonstrating that *R. pimpinellifolia* represents a prime candidate for nutraceutical exploitation.

## 1. Introduction

Rosehip (*Rosa* spp.) fruits are widely recognized as a rich source of bioactive compounds, including ascorbic acid, polyphenols, essential fatty acids, carotenoids, and organic acids, which contribute to their high antioxidant capacity and growing use in functional foods, nutraceuticals, and pharmaceutical applications [1,2]. In particular, polyphenolic compounds exhibit antioxidant activity through various mechanisms, including free radical scavenging and metal ion chelation [3,4]. These bioactive compounds have been associated with several biological activities, including anti-inflammatory, anti-oxidative, anti-mutagenic, anti-diabetic, and anti-carcinogenic effects, which further enhance the nutritional and therapeutic value of rosehip [1,2,3,5].

*Rosa* species are widely distributed across temperate and subtropical regions, exhibiting considerable ecological adaptability [5,6]. Anatolia represents one of the major centers of genetic diversity for *Rosa* species, with the eastern and central regions of Türkiye being particularly rich in native rosehip populations. This diversity contributes to substantial variation in fruit morphology and biochemical composition among genotypes [7]. Such variation provides a valuable opportunity to identify naturally occurring genotypes with distinct morphological and biochemical profiles [8].

Previous studies on wild *Rosa* genotypes have consistently demonstrated substantial variation in fruit morphology, phytochemical composition, and antioxidant capacity under different agroecological conditions. Comparative evaluations of *R. canina* and *R. dumalis* ecotypes revealed significant differences in phenolic content, antioxidant activity, and fruit quality traits, highlighting the influence of environmental and genotypic factors on their bioactive composition [7]. Similarly, morphological and biochemical characterization studies conducted on naturally growing *Rosa canina* genotypes in Türkiye have shown that such variations can be used to distinguish genotypes with favorable biochemical profiles [8]. These findings emphasize that naturally occurring variation among wild populations provides an important basis for identifying plant materials with desirable pomological and biochemical traits. Moreover, growing consumer demand for naturally derived bioactive compounds has intensified scientific interest in wild fruit species as sustainable sources of health-promoting ingredients. In this context, rosehip fruits have attracted considerable attention because of their high antioxidant capacity and rich phytochemical composition, which contribute to their nutritional quality and potential value as functional food ingredients [1,3].

In addition to genotypic variation, fruit pigmentation has also been associated with the accumulation of phenolic compounds, particularly flavonoids and anthocyanins, during fruit development and ripening [9]. These compounds contribute not only to fruit coloration but also to antioxidant activity, thereby influencing the nutritional and functional quality of rosehip fruits [3]. Recent reviews have further emphasized the importance of phenolic composition in determining the biological activity and potential functional value of rosehip fruits [1]. In addition to total phenolic measurements, the characterization of individual phenolic compounds using chromatographic techniques provides more detailed insights into fruit quality and phytochemical composition. Therefore, phenolic profiling has become an important approach for distinguishing genotypes and evaluating their potential value as food resources [10].

The antioxidant properties and phytochemical composition of *Rosa* species in Türkiye have been extensively investigated in numerous studies. One particularly relevant investigation assessed the phenolic compounds, organic acids, volatile compositions, and antioxidant activities of five *Rosa* taxa: *R. canina*, *R. dumalis*, *R. gallica*, *R. dumalis* subsp. *boissieri*, and *R. hirtissima* [11]. Nevertheless, pomological, morphological, and instrumental color traits were not evaluated in that study. Additionally, the taxa studied in the present study, *R. rugosa*, *R. villosa*, and *R. pimpinellifolia*, were excluded from that investigation with the exception of *R. canina*. While a previous study investigated a comprehensive morphological, biochemical, and molecular characterization of *R. canina* samples, including phenolic compounds and vitamin C, it was limited to a single species and did not include instrumental color measurements [8]. The wild samples of *R. canina* and *R. dumalis* were examined under various agroecological conditions [7]. Although these studies have provided valuable insights into rosehip diversity, an integrated assessment at the sample level, combining pomological traits, instrumental color measurements, biochemical properties, including antioxidant capacity and phenolic profiles, remains limited for taxonomically distinct wild *Rosa* samples collected from the same geographical area during the same harvest season. The current study addresses this gap by evaluating five wild samples from four *Rosa* taxa within a common sampling and analytical framework. Moreover, the incorporation of two geographically proximate *R. pimpinellifolia* samples enabled the evaluation of intraspecific variation as well as differences between taxonomically distinct species.

Thus, this study aimed to determine the extent of interspecific and intraspecific variation among five wild *Rosa* samples collected from Artvin by comparatively evaluating their pomological, morphological, colorimetric, biochemical, antioxidant, and individual phenolic profiles.

## 2. Results and Discussion

Considerable variation was observed among the investigated wild rosehip samples in terms of pomological, biochemical, and phenolic characteristics, indicating substantial morphological and phytochemical diversity among the investigated rosehip samples from Artvin, Türkiye. The pomological and morphological properties of the studied samples are presented in Figure 1.

Clear differences were observed among the samples in terms of fruit size, weight, and shape. RS1 (*R. rugosa*) produced the largest fruits, with an average fruit weight of 4.45 g and a flattened-spherical shape, whereas RS4 and RS5 (*R. pimpinellifolia*) were characterized by smaller and globose fruits, with average weights of 1.20 and 0.65 g, respectively. Comparable variation in pomological traits among naturally growing rosehip samples has previously been documented [7,8,12].

Biochemical properties and antioxidant activities of rosehip samples are summarized in Table 1. Total soluble solid (TSS) values differed significantly among the samples and ranged from 19.60% in RS1 to 53.83% in RS5. The TSS values obtained in the present study were generally higher than those previously reported for rosehip fruits, for which values ranging from 14 to 42.6 °Brix have been reported [13,14]. Similar genotype and environment-dependent variation in soluble solid content has also been documented in naturally growing *Rosa* ecotypes and genotypes [7,8]. In particular, the elevated TSS values observed in RS4 and RS5 may reflect species- and sample-related differences in fruit composition and metabolite accumulation.

Significant differences were observed among the samples in fruit color parameters. Hunter L*, a*, and b* values were markedly lower in RS4 and RS5 than in other samples, indicating darker fruit coloration accompanied by reduced red and yellow chromatic components in these samples (Table 1). Similar changes in color parameters have previously been reported during rosehip ripening, where decreases in L* and b* values were associated with progressive fruit darkening and compositional changes [15]. The markedly dark purplish-black coloration of RS4 and RS5 may be associated with greater anthocyanin accumulation, particularly cyanidin-3-glucoside, which has been reported as a predominant pigment in black rosehip (*R. pimpinellifolia*) [16]. Because anthocyanins are synthesized through the phenylpropanoid–flavonoid pathway [17,18], the co-occurrence of dark coloration with high TPC and TFC values in RS4 and RS5 is consistent with their elevated phenolic accumulation and antioxidant potential.

One of the most important nutritional and functional characteristics of rosehip fruits is their high ascorbic acid content. In the present study, ascorbic acid levels ranged from 78.20 to 211.41 mg/100 g FW, with the highest value detected in RS5, followed by RS4, whereas RS2 exhibited the lowest content (Table 1). These findings indicate substantial variation in vitamin C content among the samples investigated. Previous studies have similarly reported considerable variability in ascorbic acid content among different *Rosa* species and genotypes [1,11,13,19]. The higher ascorbic acid levels observed in RS4 and RS5 may have contributed to their stronger antioxidant capacity. However, because phenolic and flavonoid contents were also high in these samples, the specific contribution of ascorbic acid to the measured antioxidant activity could not be determined. As summarized in Table 2, ascorbic acid concentrations reported for rosehip fruits span a broad range across species and studies [1,11,13,19]. Nevertheless, rosehip fruits are consistently recognized as rich natural sources of vitamin C compared with many commonly consumed fruit species [1,6].

Total phenolic content (TPC) and total flavonoid content (TFC) of the wild samples are presented in Figure 2. Considerable variation was observed among the samples investigated. TPC values ranged from 905 to 2902 mg GAE/100 g FW, while TFC values varied between 115 and 510 mg QE/100 g FW. RS5 exhibited the highest TPC and TFC values, followed by RS4, whereas RS2 had the lowest levels. Similar variability in phenolic and flavonoid composition among samples has previously been reported for wild Rosa species in the literature [1,7,13]. Previous studies have also emphasized the high phenolic and antioxidant potential of black rosehip (*R. pimpinellifolia*) [13,20]. Although generally considered less common than *R. canina*, this species was found to be widely distributed within the study area examined. Its occurrence has also previously been reported in nearby northeastern regions of Türkiye, including Ardahan [13]. These elevated TPC and TFC values in RS4 and RS5 are further reflected in their antioxidant capacity, as discussed below.

Antioxidant assays also revealed considerable variation among the samples. FRAP values ranged from 1.71 to 4.39 µmol Trolox/g, with the highest reducing power observed in RS5 and RS4. Similarly, DPPH radical scavenging activity differed significantly among the samples. Since lower SC_50_ values indicate stronger DPPH radical-scavenging activity, RS5 (3.89 mg/mL) and RS4 (4.34 mg/mL) exhibited the strongest radical scavenging capacities, whereas RS2 showed the weakest activity (17.15 mg/mL) (Table 1). Previous research on black rosehip has indicated that fruit antioxidant activity may result from the contributions of multiple compounds rather than a single constituent [20]. Consistent with this interpretation, the strong FRAP and DPPH responses of RS4 and RS5 occurred together with high TPC, TFC, and ascorbic acid contents, and distinctive individual phenolic profiles, suggesting a multicomponent basis for their antioxidant capacity. Similar findings have previously been reported for *R. pimpinellifolia*, emphasizing the remarkable bioactive and antioxidant potential of this species [13,20].

**Table 2:** Total phenolic compounds, total flavonoid/flavonol compounds, and ascorbic acid contents of different wild rosehip (*Rosa* spp.) samples.

Species/Variety & Processing State	Ascorbic Acid	Total Phenolic Compounds (TPC)	Total Flavonoid Compounds (TFC)	References
*Rosa canina*	101.38 mg/100 g dw	31.08 mg GAE/g dw	9.48 mg rutin/g dw	[11]
*Rosa canina*	320–423 mg/100 g	-	-	[19]
*Rosa dumalis*	65.75 mg/100 g dw	36.86 mg GAE/g dw	10.48 mg rutin/g dw	[11]
*Rosa gallica*	160.30 mg/100 g dw	31.51 mg GAE/g dw	10.81 mg rutin/g dw	[11]
*Rosa dumalis* subsp. *boissieri*	97.68 mg/100 g dw	52.94 mg GAE/g dw	9.31 mg rutin/g dw	[11]
*Rosa hirtissima*	136.14 mg/100 g dw	35.73 mg GAE/g dw	9.65 mg rutin/g dw	[11]
*Rosa canina*	754.48 mg/100 g	6298 mg GAE/100 g	Not reported	[13]
*Rosa pimpinellifolia*	24.93 mg/100 g	1081 mg GAE/100 g	Not reported	[13]
*Rosa villosa*	119.83 mg/100 g	2944 mg GAE/100 g	Not reported	[13]
*Rosa dumalis*	254.81 mg/100 g	4411 mg GAE/100 g	Not reported	[13]
*Rosa canina*	154.6 mg/L	37.3 mg GAE/g	526.2 mg QE/L	[21]
Mixed *Rosa* spp.	605.1–791.2 mg/100 g	8654–9564 mg GAE/100 g	-	[14]
Mixed *Rosa* spp.	280.0–297.0 mg/100 g	8265–8390 mg GAE/100 g	-	[14]
*Rosa rugosa*	798–3036 mg/100 g	1009–1327 mg GAE/100 g fw	-	[1,6]
*Rosa roxburghii*	2585.0 mg/100 g	152.4–159.7 mg/100 g fw	-	[1]
*Rosa canina*	320–423 mg/100 g	-	-	[19]
*Rosa pimpinellifolia* L.	199–305 mg/100 g	-	-	[19]

(-): not studied.

**Figure 2:**
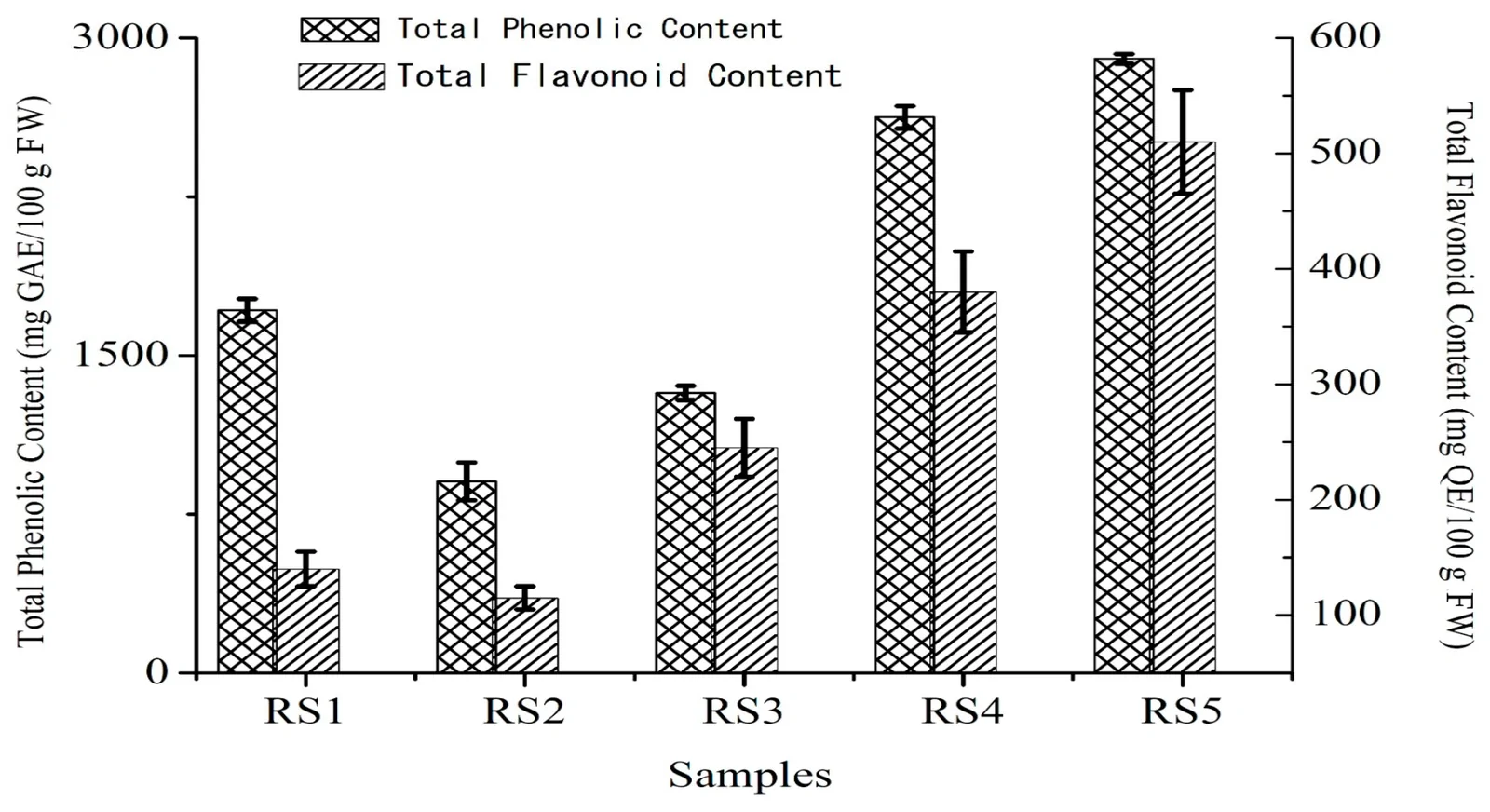
Total phenolic and flavonoid content of the wild rosehip (*Rosa* spp.) samples.

The qualitative and quantitative phenolic compositions of the rosehip samples were identified using HPLC–PDA and are summarized in Table 3. The corresponding chromatographic profiles are illustrated in Supplementary Figure S1. Of the 26 reference standards analyzed, six phenolic compounds were identified and quantified in the investigated wild rosehip samples under the applied analytical conditions. Gallic acid and ellagic acid were detected in all samples and represented the predominant phenolic acids. Gallic acid contents ranged from 0.614 to 1.816 mg/100 g FW, with the highest value observed in RS5. Ellagic acid concentrations varied from 0.29 to 1.655 mg/100 g FW, and the highest content was detected in RS2. Consistent with our findings, ellagic acid has been reported as the dominant phenolic compound in *R. canina* genotypes [21]. These compounds are recognized as important antioxidant phenolics in rosehip fruits and have previously been reported as major constituents in different *Rosa* species [1]. Marked differences in flavonoid composition were observed among the samples. Quercetin was detected only in RS1, RS2, and RS3, whereas rutin was identified in RS3, RS4, and RS5. RS5 contained a particularly high rutin concentration (20.567 mg/100 g FW). Catechin was detected only in RS4 and RS5, at concentrations of 22.533 and 28.838 mg/100 g FW, respectively, whereas epicatechin was detected exclusively in RS5 at 35.350 mg/100 g FW. Although these compounds were found in the dark purplish-black *R. pimpinellifolia* samples, catechin and epicatechin are non-pigmented flavan-3-ols and therefore cannot directly account for fruit coloration. Instead, their co-occurrence with dark pigmentation may reflect differences in metabolite distribution between the interconnected anthocyanin and flavan-3-ol branches of the flavonoid pathway [17]. However, because anthocyanin concentrations, pathway-related gene expression, and enzyme activities were not measured, this interpretation should be regarded as tentative. Overall, the HPLC–PDA results revealed substantial qualitative and quantitative differences in phenolic composition among the investigated samples. The non-detection of the remaining targeted compounds should not be interpreted as evidence of their complete absence from the samples, because extraction efficiency, compound polarity, chemical form, and interactions with matrix components may have influenced their recovery and detection [22,23]. Moreover, the targeted HPLC–PDA approach enabled the identification of compounds included in the reference-standard panel but did not comprehensively characterize other phenolic forms potentially present in the samples, including ester-bound and glycosidically bound conjugates that can be released only after alkaline or acid hydrolysis, as well as oligomeric derivatives such as procyanidins [23]. Therefore, the six quantified compounds represent the targeted phenolic profile obtained under the applied analytical conditions rather than the overall phenolic composition of the rosehip fruits. A direct comparison with the phenolic profile reported for fresh black rosehip fruits [20] is limited by differences in the analytical scopes of the two studies. In their HPLC analysis, nine phenolic compounds were identified, with chlorogenic acid being the predominant compound, followed by gallic acid. Catechin and epicatechin were not included in their analytical panel [20]. In the present study, catechin was the most abundant quantified compound in RS4, whereas catechin and epicatechin were the predominant compounds in RS5. Chlorogenic acid was included in the validated reference-standard panel but remained below the LOD in all samples. Accordingly, these apparent differences should not be interpreted as definitive evidence of contrasting phenolic compositions. Rather, they demonstrate that the composition of the reference-standard panel and the analytical scope of each method should be considered when comparing targeted HPLC phenolic profiles across studies. The high phenolic and antioxidant levels observed in RS4 and RS5 may indicate greater accumulation of phenylpropanoid- and flavonoid-derived metabolites in the investigated *R. pimpinellifolia* samples. However, the individual phenolic profiles of RS4 and RS5 differed markedly. Although both samples were identified as *R. pimpinellifolia*, RS5 contained substantially more rutin and was the only sample in which epicatechin was detected, while marked differences were also observed in gallic and ellagic acid contents. These findings demonstrate that samples belonging to the same species may exhibit substantially different phenolic compositions. Phenolic and antioxidant traits in rosehip fruits may be influenced by both genotype and local environmental conditions. A study of wild *R. canina* collected from three different locations reported significant variation in polyphenols, flavonoids, anthocyanins, and antioxidant activity both among locations and among genotypes within the same location, with genotype identified as the main determinant of the bioactive profile. More recently, an evaluation of 76 wild *R. canina* genotypes identified altitude as the primary predictor of radical-scavenging activity and latitude as a significant factor associated with TPC. Light and UV exposure, temperature, and water stress can also modify phenylpropanoid metabolism and phenolic accumulation in plants. However, the samples examined in the present study were collected from geographically close locations within a narrow altitudinal range of 1210–1260 m, and local light exposure, temperature, soil moisture, and other environmental conditions were not quantified. Therefore, the observed phytochemical differences cannot be attributed to altitude or any single ecological factor and may instead reflect the combined effects of taxonomic identity, individual plant variation, and local microenvironmental conditions. Controlled or multi-location studies are required to distinguish these effects.

In addition, anthocyanins constitute a notable portion of the secondary metabolites found in rosehips. They represent a major class of compounds responsible not only for the biological activities of the fruits but also for their distinct coloration and sensory attributes. Moreover, the qualitative and quantitative profile of these secondary metabolites, specifically their type, diversity, and concentration, is highly dependent on the intrinsic characteristics of the plant. Furthermore, extrinsic environmental factors, including climate variations, altitude, and specific geographical conditions, play a critical role in modulating their biosynthesis and accumulation. Consequently, the anthocyanin composition can fluctuate significantly across different wild samples and harvest regions. Although specific anthocyanin derivatives, such as cyanidin-3-O-glucoside, pelargonidin-3,5-di-O-glucoside, and cyanidin-3-O-rutinoside, are frequently reported as the predominant polyphenols in these fruits, they could not be quantified in the present study due to the unavailability of their respective analytical reference standards.

To further explore the relationships among the measured biochemical, colorimetric, antioxidant, and phenolic variables, an exploratory Pearson correlation and principal component analysis (PCA) were performed using the sample-level mean values reported in Table 1 and Table 3 (13 variables, *n* = 5 samples). Given the limited number of samples (*n* = 5, df = 3), this analysis should be interpreted as descriptive/exploratory rather than confirmatory. Because of the small sample size and the large number of simultaneous pairwise comparisons, correlation coefficients are reported without significance symbols and are not intended as formal hypothesis tests (Figure 3). In addition, as shown in Table 4, Pearson correlation coefficients (r) indicate the relationships among the measured variables.

To provide an exploratory overview of the relationships among the evaluated pomological, biochemical, and antioxidant parameters across the five composite rosehip samples, Principal Component Analysis (PCA) and Pearson correlation analysis were performed (Figure 3). However, given the limited sample size (*n* = 5), these analyses are presented strictly as descriptive and qualitative visualizations of the dataset rather than inferential statistical models. The PCA biplot illustrates the relative distribution and exploratory grouping of the analyzed composite extracts based on their primary chemical and physical characteristics, with the first two principal components accounting for 78.5% of the total variance. Crucially, it must be emphasized that due to the constrained sample pool and the absence of molecular authentication, this PCA model does not provide definitive evidence for the biological or taxonomic separation of the investigated *Rosa* species. Instead, it serves solely as a descriptive snapshot of the multidimensional variance observed among these specific composite sample extracts, pointing toward trends that warrant validation through broader population-scale studies.

## 3. Materials and Methods

### 3.1. Chemicals and Reagents

Methanol, ethanol, acetonitrile, ethyl acetate, diethyl ether, acetic acid, Folin–Ciocalteu reagent, sodium carbonate, ferric chloride, Trolox, 2,2-diphenyl-1-picrylhydrazyl (DPPH), 2,4,6-tris(2-pyridyl)-s-triazine (TPTZ), and sodium acetate were supplied by Sigma Aldrich (Darmstadt, Germany). Aluminum nitrate, and ammonium acetate were supplied from Merck (Darmstadt, Germany). The phenolic standards of gallic acid, protocatechuic acid, p-hydroxybenzoic acid, vanillic acid, syringic acid, caffeic acid, p-coumaric acid, ferulic acid, trans-cinnamic acid, ellagic acid, catechin, epicatechin, rutin, quercetin, myricetin, rhamnetin, galangin, chrysin, daidzein, apigenin, luteolin, pinocembrin, hesperetin, naringenin, chlorogenic acid, and caffeic acid phenethyl ester (CAPE) were purchased from Sigma–Aldrich (St. Louis, MO, USA).

### 3.2. Sample Collection and Botanical Identification

Rosehip fruit samples were collected on 15 September 2025, from the Ardanuç district of Artvin, Türkiye. Plant samples were taxonomically identified and classified based on established botanical morphological characteristics by Assoc. Prof. Dr. Hayal Akyıldırım Beğen, a botanist at Artvin Çoruh University (noting that molecular or genotypic authentication was not performed). The sample codes and corresponding morphological identifications are summarized in Table 5, while photographic documentation and morphological traits are presented in Figure 1.

### 3.3. Morphological and Pomological Characterization

Based on their morphological characteristics, each species (accession) was collected from five distinct natural shrubland habitats. To obtain a representative composite sample for each accession, mature fruits were harvested from at least five independent individual shrubs within each habitat and combined. From this representative pooled material, 21 intact fruits per accession were randomly selected and divided into three composite subgroups of seven fruits each (*n* = 3). Immediately after harvest, these subgroups were subjected to morphological and pomological evaluations. Fruit weight was measured using an analytical balance, while length and width were determined with a digital caliper. Additionally, color, general fruit morphology, and shape were documented. Following physical measurements, the seven fruits within each subgroup were homogenized together to yield three independent composite extract replicates. All subsequent biochemical parameters were analyzed in triplicate using these separate extractions, reflecting the mean compositional profile of each accession.

### 3.4. Determination of Total Soluble Solids and Color Parameters

Total soluble solids (TSS) were determined according to the AOAC [24]. Juice obtained from each rosehip sample was analyzed at 20 °C using an Abbe-3L refractometer (Tokyo, Japan), and the results were expressed as °Brix. Fruit color was evaluated using the Hunter L*, a*, and b* color system in accordance with standard colorimetric procedures [25]. Colorimetric properties of the fresh fruits were determined using a Hunter Lab colorimeter (Libshang ls173 173020085, Shenzhen, China). For each accession, measurements were directly obtained from 20 individual fruits, and the results were expressed as mean values ± standard deviations (SD).

### 3.5. Sample Preparation and Extraction

Following pomological characterization, fresh rosehip fruits were thoroughly rinsed under running tap water to remove surface impurities, followed by a gentle rinse with deionized water. The intact fruits were carefully blotted dry using soft paper towels without causing any mechanical damage or bruising to the tissues. To minimize enzymatic degradation and potential polyphenol oxidation during mechanical processing, fresh whole fruits were directly transferred into the extraction solvent rather than being pre-dried. Specifically, a 10 g portion, precisely weighed from cleaned and fresh fruit material, was combined with 100 mL of 70% aqueous methanol (*v*/*v*) and immediately homogenized using a high-speed laboratory blender until a completely homogeneous slurry was obtained. The resulting homogenate was subjected to ultrasound-assisted extraction in an ultrasonic bath at room temperature for 2 h to facilitate cell wall disruption and the release of phenolic compounds. Subsequently, the mixture was continuously agitated on an orbital shaker in the dark for 24 h to ensure complete extraction. Following extraction, the mixtures were first filtered through a clean, dry, multi-layered cotton muslin cloth (cheesecloth) to remove coarse fruit debris and insoluble pulp residues. The obtained liquid filtrate was subsequently filtered through Whatman No. 1 filter paper to achieve a clarified extract for further analyses. Calculations regarding the biochemical parameters were performed on a fresh weight (FW) 100 g fresh sample.

### 3.6. Determination of Ascorbic Acid Content

The ascorbic acid (Vitamin C) content of the methanolic extracts was determined employing a classical iodometric titration method, based on the protocol described [26]. This quantitative redox assay relies on the oxidation of ascorbic acid by iodine, which is generated in situ from the reaction between potassium iodate (KIO_3_) and potassium iodide (KI) under acidic conditions. Briefly, the sample extracts were initially diluted at an appropriate ratio (1:50, *v*/*v*) with deionized water. To this diluted solution, 1 g of KI and 5 mL of 1 M HCl were added, followed by the addition of 1 mL of freshly prepared starch indicator solution (1%, *w*/*v*). The prepared mixture was subsequently titrated against a standardized $5\times 10^{-5}\;M\;{\mathrm{KIO}}_{3}$ solution until a persistent blue-black color was observed for at least 30 s, indicating the titration endpoint. To eliminate potential background interferences and non-specific iodine consumption from reagents and the extract matrix, reagent and sample blanks were run in parallel under identical conditions and subtracted from the titration volumes. The ascorbic acid concentration was calculated based on the net titrant volume consumed and expressed as milligrams per 100 g of fresh weight (mg/100 g FW). Analytical grade L-ascorbic acid (Sigma-Aldrich, Merck, Darmstadt, Germany) was utilized as the reference standard to verify the stoichiometric accuracy and recovery of the titrimetric system across analytical triplicates (n=3).

### 3.7. Determination of Total Phenolic Content (TPC)

Total phenolic content of the methanolic extracts was determined using the Folin–Ciocalteu’s assay [27]. Initially, 680 μL of the extract was combined with 400 μL of 0.2 N Folin–Ciocalteu’s reagent. After a 3 min incubation period, 400 μL of 10% sodium carbonate was added, and the mixture was incubated for 2 h at room temperature. Following the incubation, the absorbance was measured using a spectrophotometer at a wavelength of 760 nm. A gallic acid calibration curve (y=1.2823x+0.0826) was constructed to determine the total phenolic content. The results were expressed as mg GAE/100 g FW.

### 3.8. Determination of Total Flavonoid Content (TFC)

The total flavonoid content of the methanolic extracts was quantified using a modified version of the method [28]. In this adapted procedure, aluminum nitrate (Al(NO_3_)_3_) was employed instead of aluminum chloride (AlCl_3_) because of its lower solubility in water. Briefly, 50 µL of the methanolic extract was mixed with 50 µL of 10% Al (NO_3_)_3_ and 50 µL of 1.0 M ammonium acetate (NH_4_CH_3_COO). The reaction mixture was adjusted to a final volume of 3.0 mL with 99% ethanol and incubated at 25 °C for 45 min. Following incubation, absorbance was recorded at 415 nm. Quantification was performed using a calibration curve (y=6.6167x+0.0035) prepared with six quercetin standard solutions (0.031–0.50 mg QE/100 mL). The results were calculated from this standard curve and expressed as mg QE/100 g FW.

### 3.9. Ferric Reducing Antioxidant Power (FRAP) Assay

The total antioxidant capacity of the extract was determined using the Ferric Reducing Antioxidant Power (FRAP) method [29]. The FRAP working solution was freshly prepared by mixing 300 mM sodium acetate buffer (pH 3.6), 10 mM TPTZ solution, and 20 mM FeCl_3_ solution in a volumetric ratio of 10:1:1. For the analysis, 1.5 mL of the FRAP reagent was combined with 0.05 mL of the methanolic sample extract, giving a total reaction volume of 1.55 mL. The reaction mixture was incubated at 37 °C for 4 min, and the absorbance was subsequently recorded at 595 nm. A standard calibration curve was generated using Trolox solutions ranging from 1.0 to 50 μM. The ferric reducing antioxidant power of each sample was determined from the linear regression equation of the Trolox standard curve, accounting for the extraction ratio and reaction dilution factors. Results were expressed as micromoles of Trolox equivalents per gram of fresh weight (μmol TE/g FW).

### 3.10. Determination of DPPH Radical Scavenging Activity

The free radical scavenging capacity of the extracts was evaluated using the DPPH (2,2-diphenyl-1-picrylhydrazyl) assay, as described by Molyneux (2004) [30]. In brief, 750 μL of a 100 μM DPPH solution prepared in methanol was mixed with 750 μL of the sample extract. The reaction mixture was kept at room temperature in the dark for 45 min to allow the reaction to proceed to completion. Subsequently, the absorbance was recorded at 517 nm. The results were expressed as scavenging concentration SC_50_ values (mg/mL), defined as the sample concentration required to reduce the initial DPPH radical level by 50%. Lower SC_50_ values indicate stronger radical-scavenging activity. To determine these values, individual concentration–response curves were constructed for each sample using at least six different concentrations.

### 3.11. Determination of Phenolic Profiling Using HPLC-PDA

Before conducting phenolic compound profiling of the methanolic extracts by HPLC–PDA, a liquid–liquid extraction procedure was applied to concentrate the analytes. Initially, 10 mL of the rosehip extract was evaporated at 40 °C using a rotary evaporator [31]. The remaining residue was reconstituted in 10 mL of distilled water, and the pH was adjusted to 2 with concentrated HCl. The solution was then subjected to three consecutive extractions with diethyl ether and ethyl acetate. The collected organic fractions were pooled, and after solvent evaporation, the residue was redissolved in 2 mL of methanol. The final solution was filtered through a 0.45 μm regenerated cellulose (RC) membrane filter before phenolic analysis.

The phenolic profile was determined using an HPLC–PDA system (Shimadzu LC-20AT, Shimadzu Liquid Corporation, Tokyo, Japan) equipped with a photodiode array detector and a C18 column (250 mm × 4.6 mm, 5 µm; GL Sciences, Tokyo, Japan). Quantification was achieved by constructing calibration curves with 26 individual phenolic standards [32]. The mobile phase consisted of (A) ultrapure water containing 2% acetic acid and (B) acetonitrile: water (70:30, *v*/*v*). Both standards and samples were injected at a volume of 20 µL. The chromatographic conditions included a column temperature of 30 °C and a flow rate of 1.0 mL/min [32]. A gradient elution was performed at a flow rate of 1.0 mL/min with the following time program.

- 0.00 min: 82% A and 18% B (initial composition)
- 5.00 min: 81% A and 19% B
- 10.00 min: 73% A and 27% B
- 14.00 min: 62% A and 38% B
- 25.00 min: 35% A and 65% B
- 40.00 min: 10% A and 90% B (final gradient composition)
- 40.01 min: 82% A and 18% B (rapid return to initial ratio)
- 40.01–50.00 min: 82% A and 18% B (column re-equilibration for 10 min)

The total chromatographic run time was 50 min. Both standards and samples were injected at a volume of 20 μL with the column temperature set at 30 °C and the autosampler cell maintained at 20 °C [32]. Phenolic compounds were monitored at 250, 280, 320, or 360 nm according to the spectral maximum assigned to each standard [32]. Phenolic compounds were identified by comparing their retention times and UV–Vis spectra with those of authentic reference standards analyzed under identical chromatographic conditions [32]. The detected compounds were quantified at their compound-specific wavelengths and retention times as follows: gallic acid at 280 nm (3.93 min), catechin at 280 nm (7.05 min), epicatechin at 280 nm (9.54 min), rutin at 250 nm (15.25 min), ellagic acid at 250 nm (15.50 min), and quercetin at 360 nm (23.22 min). All specific detection wavelengths and retention times, together with the comprehensive analytical validation parameters—including the coefficients of determination (R2), limits of detection (LOD), limits of quantification (LOQ), relative error, and recovery values derived from the previously validated RP-HPLC-PDA method [32], are detailed in Table S1 and Figure S1.

### 3.12. Statistics

All analyses were performed in triplicate using the three extraction replicates per accession described in Section 3.3, and the results are expressed as mean values. Because these replicates originate from a single pooled composite sample per accession rather than from independently sampled plants (Section 3.3), they quantify analytical/extraction variability rather than plant-to-plant biological variability. Data were statistically analyzed using SPSS software (26,0, IBM Corp., Armonk, NY, USA). To compare the mean values obtained for the five composite samples, one-way analysis of variance (ANOVA) was employed, followed by Tukey’s honestly significant difference (HSD) post hoc test for multiple comparisons (*p* < 0.05). These comparisons describe differences among the analyzed composite samples and should not be interpreted as formal tests of genotype- or population-level differences, since each accession was represented by a single pooled composite sample rather than by multiple independently sampled plants. Additionally, Principal Component Analysis (PCA) was applied to explore the distribution of samples and the contribution of each variable. An unrotated PCA loading matrix was extracted, and a PCA biplot on the PC1-PC2 plane was generated to simultaneously display the samples (points) and the measured variables (vectors). Pearson correlation coefficients among the measured variables were also calculated as an exploratory analysis; given the small sample size (*n* = 5) and the resulting large number of simultaneous pairwise comparisons, these correlations are reported without significance symbols and were not treated as formal hypothesis tests.

## 4. Conclusions

The present study demonstrated substantial pomological, biochemical, and phenolic variation among wild rosehip (*Rosa* spp.) samples collected from Artvin, northeastern Türkiye. Significant differences were observed among the five composite samples in fruit morphology, color parameters, total soluble solids, ascorbic acid content, total phenolic and flavonoid contents, antioxidant capacity, and targeted phenolic profiles, describing the diversity of the analyzed accessions under the composite-sampling design detailed in Section 3.3. Among the investigated samples, *R. pimpinellifolia* (RS4 and RS5) exhibited relatively high ascorbic acid, total phenolic, and flavonoid contents, together with strong FRAP and DPPH responses. HPLC-PDA analysis also revealed marked differences in individual phenolic composition, including the detection of catechin in both RS4 and RS5 and epicatechin exclusively in RS5.

These findings demonstrate that the analyzed wild rosehip composite samples from Artvin exhibit substantial phytochemical and pomological diversity. In particular, the prominent profiles observed in RS4 and RS5 support their potential for further phytochemical and bioactivity investigations. However, because taxonomic classifications in this study were established exclusively on botanical morphological criteria without molecular or genotypic verification, these variations reflect differences among the evaluated composite sample pools rather than generalized, definitive species-level chemotaxonomic traits. Therefore, future studies coupling comprehensive molecular barcoding and population-level sampling with assessments of bioaccessibility, bioavailability, safety, processing stability, and in vivo bioactivity are warranted. Such integrative approaches will both validate taxon-specific distinctions and clarify the functional food, nutraceutical, and conservation potential of naturally occurring rosehip germplasm.

## Figures and Tables

**Figure 1:**
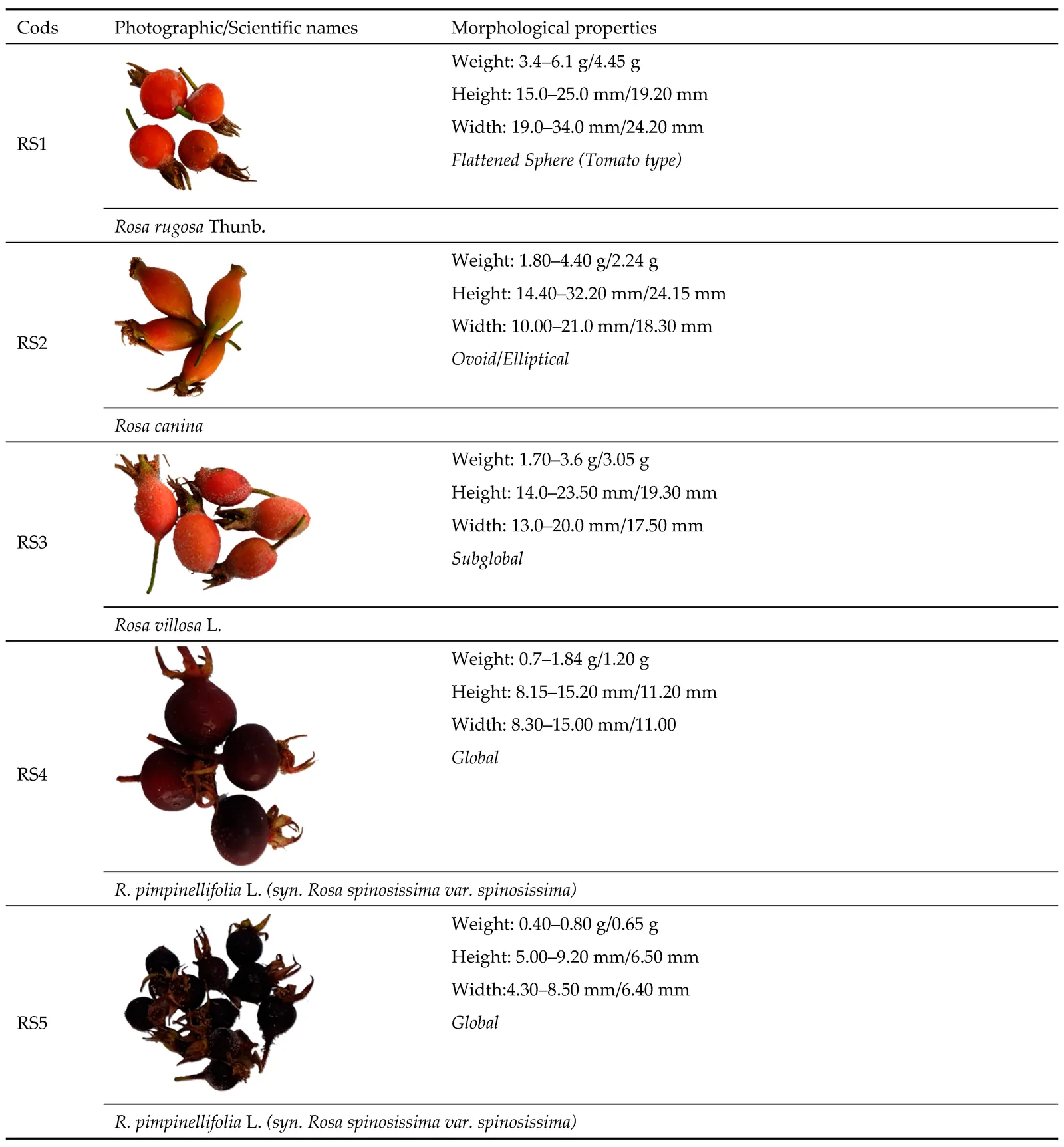
Pomological and morphological characteristics of wild rosehip (*Rosa* spp.) samples.

**Figure 3:**
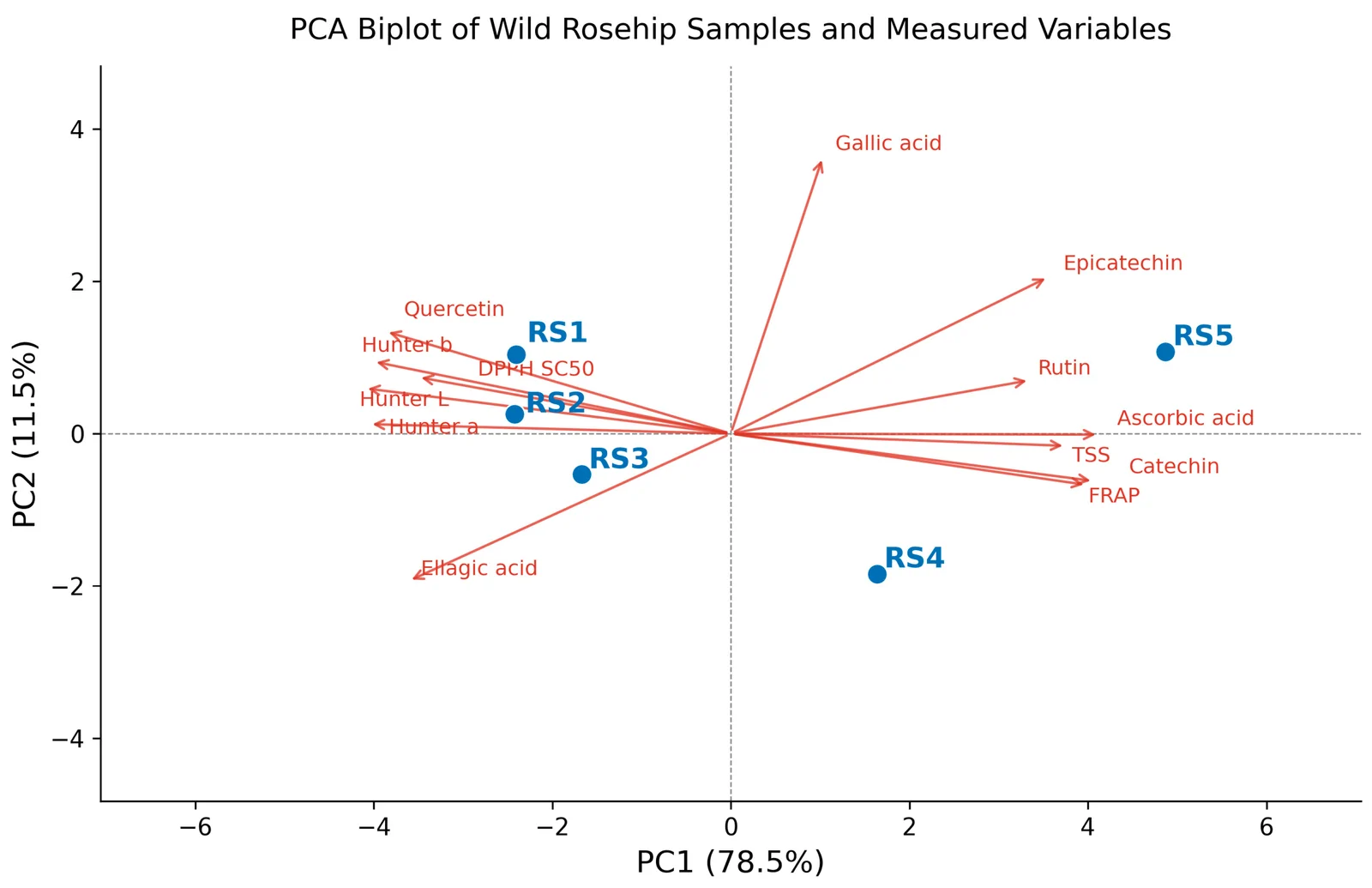
PCA biplot showing the distribution of samples (blue points) and variables (red vectors) on the PC1-PC2 plane.

**Table 1:** Biochemical properties, color parameters, and antioxidant capacities of wild rosehip (*Rosa* spp.) samples collected from Artvin, Türkiye.

Parameters	RS1	RS2	RS3	RS4	RS5
**TSS (Brix%)**	19.60 ± 0.65 ^a^	32.44 ± 1.05 ^b^	34.32 ± 1.14 ^b^	38.41 ± 1.12 ^c^	53.83 ± 1.26 ^d^
**Color/Hunter L**	35.29 ± 2.50 ^a^	31.69 ± 2.80 ^a^	31.78 ± 3.50 ^a^	11.58 ± 4.50 ^b^	3.19 ± 2.00 ^c^
**Color/Hunter a**	54.97 ± 3.10 ^a^	41.79 ± 3.2 ^b^	48.45 ± 4.00 ^b^	23.09 ± 5.00 ^c^	3.26 ± 2.52 ^d^
**Color/Hunter b**	49.04 ± 3.03 ^a^	37.11 ± 3.24 ^a^	36.61 ± 4.51 ^a^	10.88 ± 5.0 ^b^	1.15 ± 2.50 ^c^
**Ascorbic acid (mg/100 g FW)**	102.25 ± 5.06 ^c^	78.20 ± 3.34 ^d^	103.01 ± 7.58 ^c^	163 ± 6.33 ^b^	211.41 ± 13.84 ^a^
**FRAP (µmol Trolox/g FW)**	2.23 ± 0.35 ^b^	1.71 ± 0.16 ^b^	2.05 ± 0.09 ^b^	4.00 ± 0.35 ^a^	4.39 ± 0.60 ^a^
**DPPH SC_50_ (mg/mL)**	10.01 ± 1.57 ^b^	17.15 ± 1.61 ^a^	11.67 ± 0.51 ^b^	4.34 ± 0.38 ^c^	3.89 ± 0.53 ^c^

^a^, ^b^, ^c^, ^d^ letters in each cell indicate differences among the five composite samples at *p* < 0.05 (one-way ANOVA followed by Tukey’s HSD test; replicate structure described in Section 3.12).

**Table 3:** Phenolic profiling (mean ± SD, *n* = 3) of the wild rosehip (*Rosa* spp.) samples determined by HPLC-PDA (mg/100 g FW).

Phenolics (mg/100 g FW)	RS1	RS2	RS3	RS4	RS5
Gallic acid	1.73 ± 0.05 ^a^	1.35 ± 0.06 ^b^	0.61 ± 0.02 ^c^	0.80 ± 0.05 ^d^	1.82 ± 0.05 ^a^
Ellagic acid	1.29 ± 0.01 ^a^	1.66 ± 0.01 ^b^	1.43 ± 0.04 ^c^	1.37 ± 0.01 ^c^	0.29 ± 0.01 ^d^
Quercetin	0.84 ± 0.01 ^a^	0.71 ± 0.02 ^b^	0.74 ± 0.01 ^b^	<LOD	<LOD
Rutin	<LOD	<LOD	11.20 ± 0.06 ^a^	4.89 ± 0.10 ^b^	20.57 ± 0.12 ^c^
Epicatechin	<LOD	<LOD	<LOD	<LOD	35.35 ± 0.13
Catechin	<LOD	<LOD	<LOD	22.53 ± 0.21 ^a^	28.84 ± 0.03 ^b^

*p*-OH Benzoic acid, Protocatechuic acid, Syringic acid, Vanillic acid, t-Cinnamic acid, Ferulic acid, p-Coumaric acid, Caffeic acid, CAPE, Rhamnetin, Myricetin, Galangin, Chrysin, Daidzein, Apigenin, Luteolin, Pinocembrin, Hesperetin, Naringenin, and chlorogenic acid were below the LOD. ^a^, ^b^, ^c^, ^d^, Letters in each column indicate differences among the five composite samples at *p* < 0.05 (one-way ANOVA followed by Tukey’s HSD test; replicate structure described in Section 3.12).

**Table 4:** Pearson correlation coefficients (*r*) among the measured variables.

	TSS	Hunter L	Hunter a	Hunter b	Ascorbic Acid	FRAP	DPPH	Gallic Acid	Ellagic Acid	Quercetin	Rutin	Epicatechin	Catechin
**TSS**	1	-	-	-	-	-	-	-	-	-	-	-	-
**Hunter_L**	−0.89	1	-	-	-	-	-	-	-	-	-	-	-
**Hunter_a**	−0.93	0.98	1	-	-	-	-	-	-	-	-	-	-
**Hunter_b**	−0.92	0.99	0.98	1	-	-	-	-	-	-	-	-	-
**Ascorbic acid**	0.83	−0.96	−0.92	−0.92	1	-	-	-	-	-	-	-	-
**FRAP**	0.76	−0.97	−0.90	−0.93	0.98	1	-	-	-	-	-	-	-
**DPPH SC_50_**	−0.56	0.83	0.71	0.76	−0.91	−0.94	1.0	-	-	-	-	-	-
**Gallic acid**	0.07	−0.16	−0.24	−0.04	0.25	0.17	−0.1	1.0	-	-	-	-	-
**Ellagic acid**	−0.74	0.76	0.78	0.70	−0.87	−0.76	0.7	−0.57	1	-	-	-	-
**Quercetin**	−0.81	0.98	0.93	0.97	−0.91	−0.96	0.83	−0.03	0.62	1	-	-	-
**Rutin**	0.86	−0.71	−0.73	−0.72	0.78	0.64	−0.59	0.1	−0.84	−0.58	1.0	-	-
**Epicatechin**	0.82	−0.76	−0.82	−0.72	0.82	0.69	−0.56	0.57	−0.97	−0.61	0.85	1.0	-
**Catechin**	0.83	−0.99	−0.96	−0.97	0.97	0.99	−0.87	0.18	−0.75	−0.98	0.66	0.73	1.0

Given the small sample size (*n* = 5 accessions, df = 3) and the large number of simultaneous pairwise comparisons (78 correlations), conventional significance testing was not considered statistically robust; correlation coefficients are therefore presented without significance symbols and should be interpreted as descriptive/exploratory measures of association rather than formal hypothesis tests.

**Table 5:** Sample codes, taxonomic identification, and collection information of wild rosehip samples.

Sample Code	Taxonomic İdentification	Coordinates		Approximate Elevation (m)
		Approximate Latitude (°N)	Approximate Longitude (°E)	
RS1	*Rosa rugosa* Thunb.	41.1188	42.0655	1210
RS2	*Rosa canina* L.	41.1210	42.0691	1230
RS3	*Rosa villosa* L.	41.1222	42.0676	1240
RS4	*Rosa pimpinellifolia* L.	41.1243	42.0695	1240
RS5	*Rosa pimpinellifolia* L.	41.1252	42.0662	1260

Taxonomic identification was performed at the species level; therefore, no subspecies designation was assigned. *Rosa spinosissima* var. *spinosissima* is reported as a synonym of *Rosa pimpinellifolia*. RS4 and RS5 represent two separately collected samples identified as the same species.

## Data Availability

The data supporting the findings of this study are available from the corresponding author upon reasonable request.

## Supplementary Materials

The following supporting information can be downloaded at: https://www.mdpi.com/article/10.3390/molecules31193429/s1, Table S1: Detection wavelengths, retention times, and validation parameters of the phenolic standards used in the RP-HPLC–PDA analysis. Figure S1: The chromatograms of the samples obtained by RP-HPLC-PDA are provided.

## Abbreviations

The following abbreviations are used in this manuscript:

TPC	Total Phenolic Content
TFC	Total Flavonoid Content
FRAP	Ferric Reducing Antioxidant Power
DPPH	2,2-Diphenyl-1-picrylhydrazyl
RS	Rosehip
SC_50_	Radical Scavenging Concentrations
