# Supplementary material for: Comprehensive Phenolic Profiling, Antioxidant Bioactivity, and Pomological Evaluation of Wild Rosehip (Rosa spp.) Samples
Source: Molecules. 2026 Sep 26;31(19):3429. doi: 10.3390/molecules31193429 (PMC13666180; doi:10.3390/molecules31193429)
Supplement: Supplementary file 1 [file molecules-31-03429-s001.zip › molecules-4547191-supplementary -correct.pdf]

Supplementary Materials

Comprehensive Phenolic Profiling, Antioxidant Bioactivity, and Pomological Evaluation of Wild Rosehip (*Rosa* spp.) Samples

Table S1. Detection conditions and analytical validation parameters of the RP-HPLC–PDA method used for phenolic analysis.

Standard compound	Detection wavelength (nm)	Retention time (min)	R^2	LOD (µg/mL)	LOQ (µg/mL)	Relative error	Recovery (%)
Gallic acid	280	3.93	0.999	0.043	0.142	0.007	99.296
Protocatechuic acid	250	5.70	0.999	0.062	0.205	0.008	100.838
Chlorogenic acid	320	6.68	0.999	0.034	0.115	0.018	101.757
Catechin (catechin hydrate standard)	280	7.05	0.999	0.019	0.063	0.011	98.917
p-Hydroxybenzoic acid	250	9.03	0.999	0.044	0.148	0.028	102.816
Epicatechin	280	9.54	0.997	0.033	0.109	0.028	102.843
Caffeic acid	320	10.12	0.999	0.072	0.239	0.027	102.716
Vanillic acid	250	10.36	0.998	0.043	0.143	0.022	97.803
Syringic acid	280	10.61	0.999	0.021	0.071	0.013	98.725
p-Coumaric acid	320	15.02	0.999	0.064	0.214	0.028	102.830
Rutin	250	15.25	0.999	0.047	0.156	0.004	100.417
Ellagic acid	250	15.50	0.999	0.062	0.208	0.004	99.554
Ferulic acid	320	16.26	0.995	0.026	0.088	0.031	103.073
Myricetin	360	19.53	0.998	0.032	0.108	0.013	101.348
Daidzein	250	21.86	0.999	0.050	0.166	0.007	100.719
Luteolin	360	23.06	0.994	0.032	0.106	0.034	103.351
Quercetin	360	23.22	0.999	0.029	0.098	0.009	99.101
trans-Cinnamic acid	280	24.39	0.999	0.021	0.071	0.008	99.242
Naringenin	280	25.95	0.996	0.026	0.087	0.008	99.181
Apigenin	320	26.14	0.999	0.037	0.123	0.040	103.948
Hesperetin	280	26.84	0.999	0.023	0.075	0.013	101.289
Rhamnetin	360	29.79	0.996	0.028	0.094	0.014	101.445
Chrysin	280	33.23	0.999	0.027	0.091	0.009	99.146
Pinocembrin	280	33.83	0.999	0.037	0.123	0.007	99.303
Caffeic acid phenethyl ester (CAPE)	320	34.07	0.999	0.025	0.083	0.035	103.456
Galangin	360	34.32	0.999	0.023	0.077	0.036	103.647

Note: The validation parameters (R^2, LOD, LOQ, relative error, and recovery) were obtained from the previously validated RP-HPLC–PDA method applied in the present study [32].

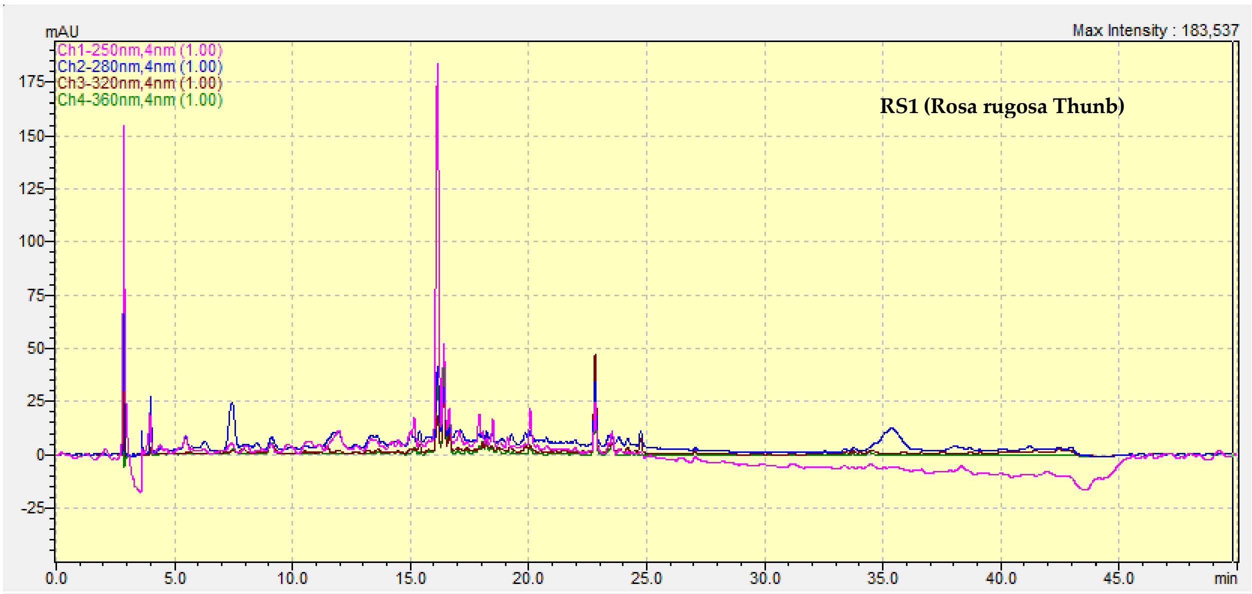
mAU
Max Intensity : 183,537
Ch1-250nm,4nm (1.00)
Ch2-280nm,4nm (1.00)
Ch3-320nm,4nm (1.00)
Ch4-360nm,4nm (1.00)
RS1 (Rosa rugosa Thunb)
min

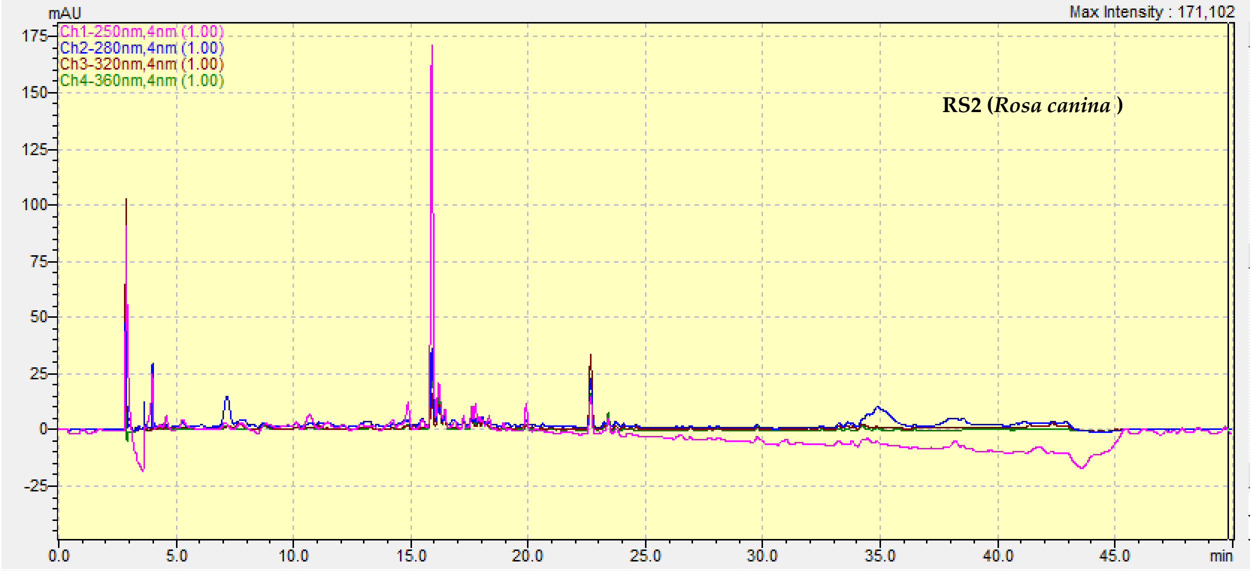
mAU
Max Intensity : 171,102
Ch1-250nm,4nm (1.00)
Ch2-280nm,4nm (1.00)
Ch3-320nm,4nm (1.00)
Ch4-360nm,4nm (1.00)
RS2 (Rosa canina)
min

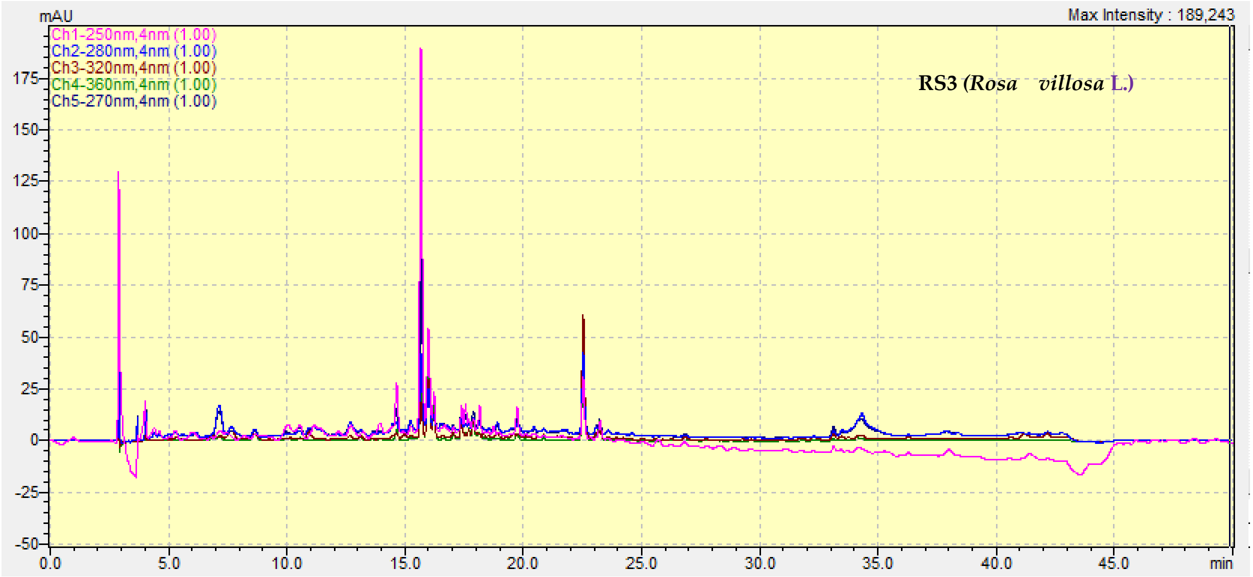
mAU
Max Intensity : 189,243
Ch1-250nm,4nm (1.00)
Ch2-280nm,4nm (1.00)
Ch3-320nm,4nm (1.00)
Ch4-360nm,4nm (1.00)
Ch5-270nm,4nm (1.00)
RS3 (Rosa villosa L.)
min

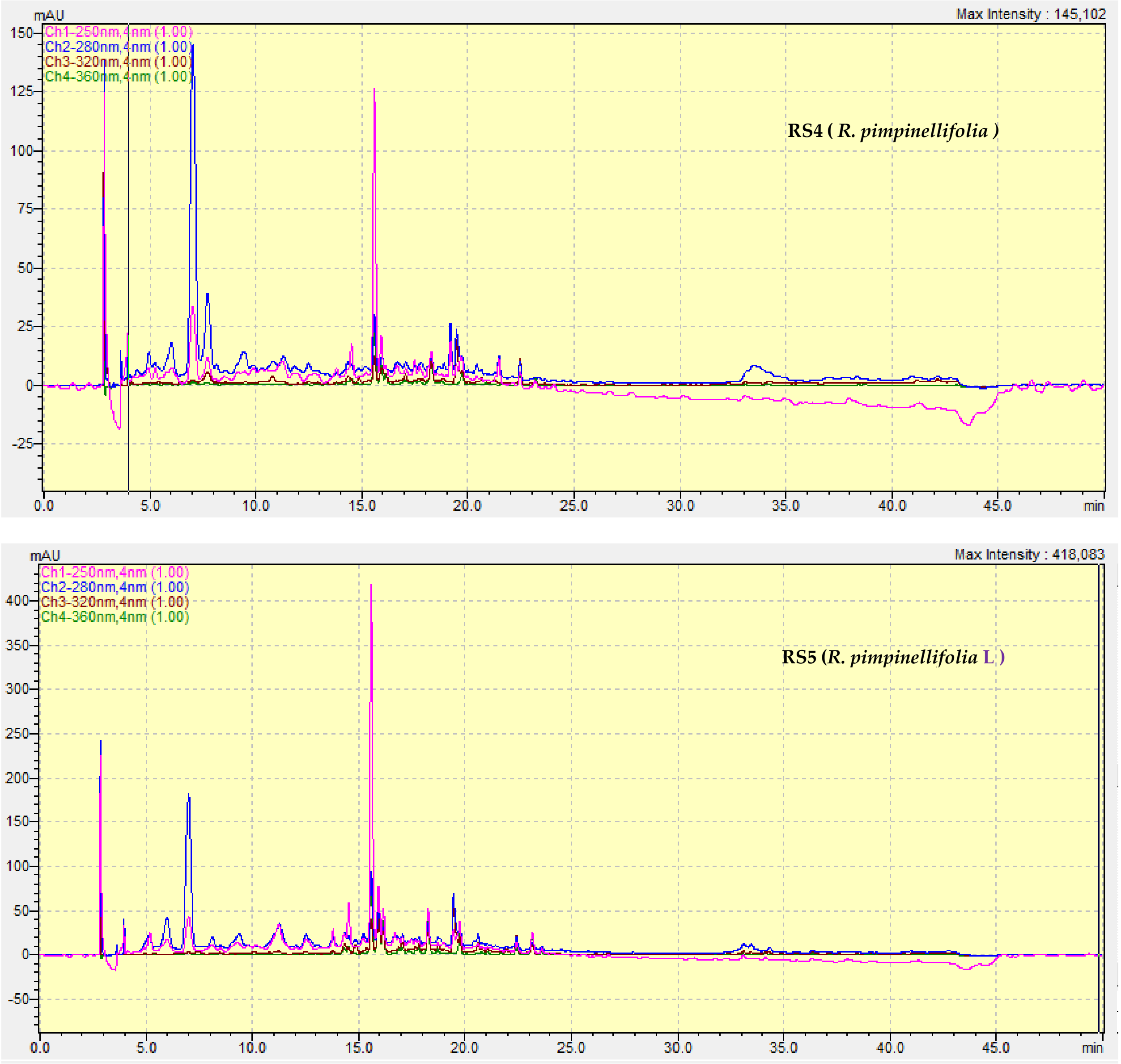

Figure S1. HPLC-PDA chromatograms of rosehip samples
